# Antimicrobial Activities and Mode of Flavonoid Actions

**DOI:** 10.3390/antibiotics12020225

**Published:** 2023-01-20

**Authors:** Amal Thebti, Ahmed Meddeb, Issam Ben Salem, Coulibaly Bakary, Sami Ayari, Farhat Rezgui, Khadija Essafi-Benkhadir, Abdellatif Boudabous, Hadda-Imene Ouzari

**Affiliations:** 1 Laboratory of Microorganisms and Active Biomolecules, Department of Biology, LR03ES03, Faculty of Sciences of Tunis, University of Tunis-El Manar, El Manar I, Tunis 2092, Tunisia; 2 Laboratory of Structural Organic Chemistry and Macromolecular, LR99ES14, University of Tunis-El Manar, El-Manar I, Tunis 2092, Tunisia; 3 Laboratory of Microbiology and LNR-Mycology, University Hospital of Abderahman Mami, Ariana 2038, Tunisia; 4 Environment and Agrifood Laboratory (ENVAL), Contaminant Group, Abidjan 21 BP 950, Côte d’Ivoire; 5 Laboratory of Molecular Epidemiology and Experimental Pathology, LR16IPT04, Pasteur Institute of Tunis, University of Tunis El-Manar, Tunis 1002, Tunisia

**Keywords:** antibacterial activity, antifungal activity, flavonoids, permeabilization

## Abstract

The emergence of antibiotics-resistant bacteria has been a serious concern for medical professionals over the last decade. Therefore, developing new and effective antimicrobials with modified or different modes of action is a continuing imperative. In this context, our study focuses on evaluating the antimicrobial activity of different chemically synthesized flavonoids (FLAV) to guide the chemical synthesis of effective antimicrobial molecules. A set of 12 synthesized molecules (4 chalcones, 4 flavones and 4 flavanones), bearing substitutions with chlorine and bromine groups at the C6′ position and methoxy group at the C4′ position of the B-ring were evaluated for antimicrobial activity toward 9 strains of Gram-positive and Gram-negative bacteria and 3 fungal strains. Our findings showed that most tested FLAV exhibited moderate to high antibacterial activity, particularly against *Staphylococcus aureus* with minimum inhibitory concentrations (MIC) between the range of 31.25 and 125 μg/mL and that chalcones were more efficient than flavones and flavanones. The examined compounds were also active against the tested fungi with a strong structure-activity relationship (SAR). Interestingly, leakage measurements of the absorbent material at 260 nm and scanning electron microscopy (SEM) demonstrated that the brominated chalcone induced a significant membrane permeabilization of *S. aureus*.

## 1. Introduction

The treatment of infectious diseases is seriously threatened by the emergence of human pathogens microorganisms resistant to the main classes of antibiotics (ATB), through the uncontrolled use of antimicrobial drugs [1,2]. Indeed, several epidemiological studies have shown a direct correlation between ATB consumption, emergence and dissemination of resistant bacteria [3] such as Methicillin-resistant *S. aureus* (MRSA) which is the second leading cause of health care-related infections [4]. Recently, increasing rates of vancomycin resistant *Enterococcus faecium* (VRE) have been reported worldwide, posing a problem as only few second-line ATB are available [5]. Bacterial strains such as MRSA, penicillin-resistant *Streptococcus pneumonia* PRSP, and VRE and multidrug-resistant (MDR) bacteria [6,7] are just few examples that have made research intonew and effective antimicrobial substances with modified or different modes of action among the top priorities [8,9]. Resistance to fluoroquinolones has also been demonstrated in *Escherichia coli* by inactivation, target protection by DNA-binding proteins, increased efflux, and target alteration [10]. In addition, a plasmid carried gene, *mcr*-1, conferring polymixins resistance was detected in Enterobacteriaceae, in China in 2015 and spread the worldwide [11]. Moreover, Carbapenem-resistant Enterobacteriaceae are resistant to most ATB and also pose a severe public health threat that requires action [12]. In this context, the chemistry of FLAV is of particular interest since they have exhibited effective antimicrobial activity against a wide range of microorganisms [13]. Chalcones are essential intermediate compounds in FLAV biosynthesis, presenting a benzal-acetophenone fundamental core. Several reports have documented the biological properties of natural or synthesized chalcones including anti-inflammatory, antitumoral, antifungal, and antibacterial activities [14]. Licochalcone A, a natural hydroxylated compound, has been shown to possess activity against Gram-positive bacteria [15]. Boubakeur et al., 2015, reported an antibacterial activity of Synthetic 7-[3-N-(1,2-O-isopropylidene-a-D-xylofuranos-5-yl)-amino-2-hydroxypropoxyl]-8-ethylflavone against *S. aureus* and *E.coli* [16]. Moreover, the inhibitory activity of quercetin, apigenin, and 3, 6, 7, 3′, 4′-pentahydroxyflavone was demonstrated against *E. coli* DNA gyrase [17]. Furthermore, the molecular modeling studies of a series of new dehydroacetic acid chalcone-1,2,3-triazole hybrids against bacterial strains (*E. coli*, *Pseudomonas aeruginosa*, *S. epidermidis*, *Bacillus subtilis*, *Aspergillus niger* and *Candida albicans*) showed that the synthetized compounds inhibited the DNA gyrase activity [18]. Ayman et al., 2018, revealed that chalcone-based dithiocarbamate effectively bind to a phosphoethanolaminetransferase involved in the resistance mechanism of *Klebsiella pneumoniae* and *P. aeruginosa* [19]. In addition, some new fluorinated chalcone-1,2,3-triazoles were reported by (Yadav et al., 2018) to interact with DNA topoisomerase [20]. Naringenin and sophoraflavanone G also showed high antibacterial activity against MRSA and streptococci by altering the membrane fluidity in the hydrophilic and hydrophobic regions [21]. Moreover, the antibiofilm activities of isovitexin and 5,7,40 -trihydroxyflavanol were reported against *S. aureus* ATCC 29213 [22]. The biological activity of FLAV depends on the chemical structure, the configuration, the total number of hydroxyl groups, and the functional groups substitutions. Antibacterial FLAV may have multiple cellular targets, rather than a specific site of action. One of their molecular actions is to form a complex with proteins by non-specific forces such as hydrogen bonding and hydrophobic effects, as well as covalent bonds formation. Thus, their antimicrobial mode of action may be related to their ability to inactivate microbial adhesins, enzymes, and cell envelope transport proteins. Results described by Mori et al., 1987 [23], suggest that the FLAV B-ring may intercalate or form a hydrogen bond with the stack of nucleic acid bases and lead to the inhibition of DNA and RNA synthesis. Gupta et al., 2019, described a derivative of isoliquiritigenin as an antibacterial drug against *S. aureus* by inhibiting the multidrug efflux pump, NorA [24]. Other compounds such as naringin and sophoraflavanone G act by altering membrane fluidity in the hydrophilic and hydrophobic regions of MRSA and streptococci. Lipophilic FLAV can also disrupt microbial membranes [25].

As we have been interested in the search for new natural and chemically synthesized molecules with antimicrobial power and because FLAV has shown an extensive range of biological activities, this study was designed to evaluate the antimicrobial activities of a set of chemically synthesized FLAV and to study the mode of action of the efficient molecules.

## 2. Results and Discussion

**Figure 1:**
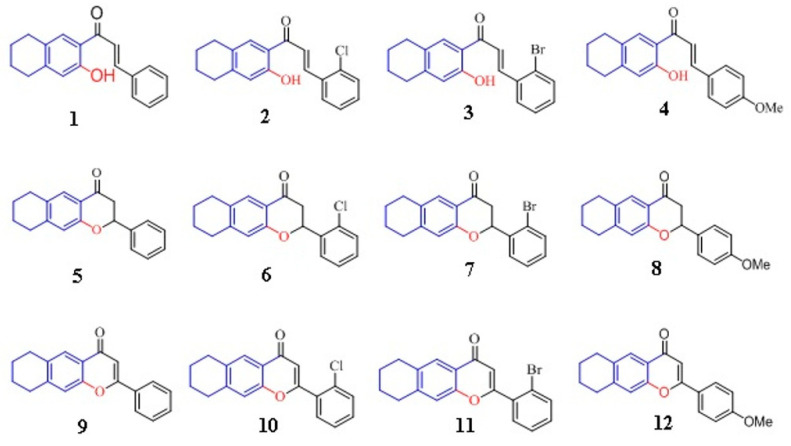
Flavonoids structures (chalcones (**1**–**4**), flavanones (**5**–**8**), and flavones (**9**–**12**)).

In the present study, we evaluated the antibacterial and antifungal activities of a series of chemically synthesized FLAV (Figure 1) by the broth microdilution method using Biolog Phenotype Microarrays. Their MICs as well as their minimal bactericidal (MBCs) or fungicidal concentrations (MFCs) were determined against different Gram-negative (*E. coli* ATCC 8739, *Salmonella* spp IPT13 and *P. aeruginosa*) and Gram-positive bacteria (*S. aureus* ATCC 25923, *S. aureus* ATCC 6538, *S. aureus* 39 MRSA, Methicillin Sensitive *S.aureus* 60 (MSSA), *E. faecium* ATCC 19436 and *Bacillus cereus* ATCC 11778) as well as some fungal species (*A. niger*, *Aspergillus flavus* and *Penicillium expansum*). Potent FLAV were evaluated for their effect on the membrane permeability of *S. aureus* 60 and *E. coli* ATCC 8739.

### 2.1. Antimicrobial Activity of Flavonoids

#### 2.1.1. Antibacterial Activity

In previous works dealing with SAR of FLAV, it was demonstrated that such derivatives displayed interesting biological activities, especially when their B-ring is diversely substituted with R = F, Cl, Br, OH, OMe [26,27,28]. Therefore, we have prepared a series of FLAV with a B-ring bearing R = Cl, Br, OMe in order to investigate their antimicrobial activity.

Thus, a series of 12 FLAV (4 chalcones, 4 flavanones, and 4 flavanones) bearing substitutions with the chlorine and bromine groups at C6′ position and the methoxy group at C4′ position of the B ring were studied for their antibacterial and antifungal activities. Results showed that most tested compounds exhibited moderate to high antibacterial activity against Gram-negative (Table 1) and Gram-positive bacteria (Table 2), particularly against S. aureus with MIC values ranging from 31.25 to 125 μg/mL. Chalcones were found to induce the highest inhibitory effect of bacterial growth compared to flavones and flavanones. Our results are in agreement with those reported by Alcara et al., 2000 [29] who found that chalcones were more active than flavanones against MRSA strains.

Our data showed also that B-ring substitutions improved the antibacterial potency. In fact, the SAR study showed that the tested chalcones induced better activity against *P. aeruginosa*, *E. faecium* ATCC 19436, MRSA 39, and *B. cereus* ATCC 11778 with lower MIC values compared to Ampicillin. Halogenated compounds are the most active molecule against Gram-positive bacteria. Sahu et al., 2012 [30] reviewed the need of hydroxyl group at the C4 position and the importance of lipophilic groups in the molecular structure. Moreover, the presence of the electron withdrawing groups was considered to be a positive factor for antibacterial activity, as they have the ability to accept hydrogen bonds. When bonded to a molecule, these atoms can interact with other molecules through hydrogen bonding. This fact has also been reported for other biological activities of chalcones derivatives, including their evaluation as antimalarials [31] and interleukin (IL)-5 inhibitors [32].

By analyzing chalcones SAR, Tran et al., 2012 [33] found that compounds with hydroxyl group at the C2- or C4-positions in B-ring showed antibacterial activity, while the hydroxyl group at the C2′-position in the A ring is not necessary for activity against MSSA and MRSA strains. It was also noticed that aromatic B-ring replacement with an heterocyclic ring containing nitrogen, oxygen, or sulfur atoms does not significantly increase the antibacterial activity [34]. In addition, the SAR study of synthesized bischalcone revealed that compounds containing free hydroxyl groups were more active than those containing methoxy groups derivatives and that the presence of electron-withdrawing groups such as chlorine increased the antibacterial activity [35].

The chlorinated compound **10** with a concentration of 31.25 μg/mL was the most active against *Staphylococcus* strains: *S. aureus* ATCC 6538, *S. aureus* 60 and *S. aureus* ATCC 25923, followed by compounds **12** and **11**, respectively, against Gram-positive bacteria. Similar results were obtained by Bahrin et al., 2016a [36], which explained the antimicrobial activity of a series of halogen-substituted flavones by the presence of a flavone moiety as well as B-ring substitutions, in particular, the bromine, chlorine and methoxy groups.

Among the tested flavanones, the methoxy-substituted **8** was the most active compound against *S. aureus* 60 and *S. aureus* ATCC 25923 with MIC values ranging from 15.62 to 62.50 μg/mL, respectively. It was more active against *P. aeruginosa* and *B. cereus* ATCC 11778 than the standard ATB with MIC values of 125 μg/mL and 62.5 μg/mL, respectively. Thus, the methoxy group substitution enhanced the compound activity compared to chlorine and bromine groups. The role of the halogen group on the antibacterial activity of FLAV was studied by Bahrin et al., 2016b [37], who reported significant inhibitory properties against Gram-positive and Gram-negative pathogens, suggesting that size is the main factor for power change rather than electronic polarity.

The MBC test results showed that the screened FLAV were bactericidal against *E. coli* ATCC 8739 but had bacteriostatic activity towards *P. aeruginosa* and *Salmonella* spp IPT13. For the Gram-positive bacteria, chalcones **2**, **3** and flavanone **12** showed bactericidal effect against *S. aureus* 60. The other FLAV were bacteriostatic.

The MIC and MBC results highlighted the importance of the antibacterial activity of these FLAV compounds which could be considered good candidate drugs to combat bacterial MRSA and VRE resistance.

#### 2.1.2. Antifungal Activity

Our results also showed that chalcones were the most potent compounds endowed with better activity compared to the reference antifungal, followed by flavanones and flavones, respectively (Table 3).

MIC values were between 7.81 and 31.25 µg/mL. In addition, the brominated chalcone **3** was the most active compound and *A*. *flavus* was the most sensitive strain. Our findings also highlighted that the flavanones exhibited a significant antifungal activity, in particular against *A*. *flavus,* with MIC values ranged between 7.81 and 31.25 μg/mL and that the compound **7** carrying a bromine group on B-ring was the most active. Among the different flavones, **11** (bearing also a bromine atom substitution) has been shown to have the highest antifungal activities at a lower MIC value than the Fluconazole against *A*. *niger*. These results showed that tested FLAV, especially chalcones, are active molecules with promising features to combat *A. flavus* and *A. niger* related infections.

Several studies have reported the antifungal activity of halogenated FLAV. Bernini et al., 2016 [38], highlighted the antifungal effect of a series of brominated and chlorinated FLAV belonging to different classes (flavanones, flavones, and catechins). Another series of mono- and di-halogenated FLAV showed remarkable antifungal activity against human pathogens, such as *Trichophyton longifus*, *C. albicans*, *Microsporium canis*, *Fusarium solanii,* and *Candida glabrata.* The most active compound was the chalcone bearing a chlorine atom on the A-ring and a bromine atom on the B-ring [39].

Another factor interfering with the compound activity is the halogen atom size. The smaller the size of the halogen atom, the higher is the inhibition percentage against *T*. *longifus*. Indeed, it has been also shown that B-ring halogenated chalcones showed the greatest fungal growth inhibition [40]. Nowakowska et al., 2007 [15], suggested that substitutions by electron donating groups on A-ring reduced chalcones activity. The enone bond in the chalcone is thought to bind to the thiol groups of proteins, which in turn leads to the yeast cell wall inhibition.

### 2.2. Lipophilicity: LogP, CrippenLogP (CLogP)

Moderate to high lipophilicity is important for the compounds’ bioactivity, allowing better biomembrane interaction or absorption. Moreover, increasing lipophilicity can improve the antimicrobial properties. To further explain the antimicrobial activity of the tested compounds, their lipophilicity (CLogP) was calculated by ChemDraw Professional 15.1 software and the results are presented in Table 4.

The CLogP values of FLAV ranged between 5.51 and 5.90 for chalcones, 4.96 and 6.24 for flavanones, and between 5.02 and 6.39 for flavanones, showing a better lipophilicity which could explain their antimicrobial activities. Vasanthanathan et al., 2006 [41], also reported a link between hydrophobicity and compound activity in the quantitative SAR of *Aspergillus* species (*A. fumigatus* and *A. niger*). Indeed, our result is concordant with the study of Vasanthanathan et al., 2006 [41], since the tested FLAV have important CLogP values and therefore better lipophilicity.

SAR results were analyzed following a comparison of the MIC results according to the chemical structures and substitution groups of the tested molecules, which showed that chalcones are more potent than flavanones and flavones, suggesting that the free hydroxyl group on C2is more important for the antimicrobial activity. Halogenated atoms, bromine, and chlorine at C6′improved the activity against Gram-positive *S. aureus* including MRSA, suggesting the importance of electron withdrawing groups for the antimicrobial activity of FLAV at those positions. The presence of methoxy group at C4′ also increased chalcones activity, most likely due to its lipophilic character, allowing better interaction with bacterial membrane. The activity of flavanones is more important for halogenated compounds against MRSA. However, substitution with the methoxy group on C4′ seems more important against the other tested Gram-positive bacteria; *S. aureus*, *E. faecium,* and *B. cereus*. Chlorinated flavones at C6′ also inhibited the growth of *S. aureus* at lower MIC values (31.25 μg/mL) and substitution with methoxy group at C4′ inhibited the growth of MRSA at the concentration of 62.5 μg/mL. Likewise, chalcones are more potent than flavanones and flavones against fungi, and the results highlighted the importance of the presence of free hydroxyl group and also bromine atom at positions C2 and C6′, respectively. These results could contribute to enrich the pharmacological features regarding the antimicrobial activity of FLAV.

### 2.3. Mode of Flavonoids Actions

The FLAV effect on the bacteria membrane was carried out by measuring the material release at 260 nm, the number of viable bacteria, and by nucleic acids UV visualization for two bacteria, *S. aureus* 60 and *E. coli* ATCC 8739, during 180 min and after 24 h. SEM analyses were also performed. 

#### 2.3.1. Leakage of 260 nm Absorbing Material for *S. aureus*

Suspensions of washed bacteria (*S. aureus* 60) were treated with (4 × [MIC]) of the most active FLAV; chalcone **3** (125 μg/mL), flavone **10** (125 μg/mL), flavanone **8** (62.5 μg/mL) and Amp (15.6 μg/mL). 

**Figure 2:**
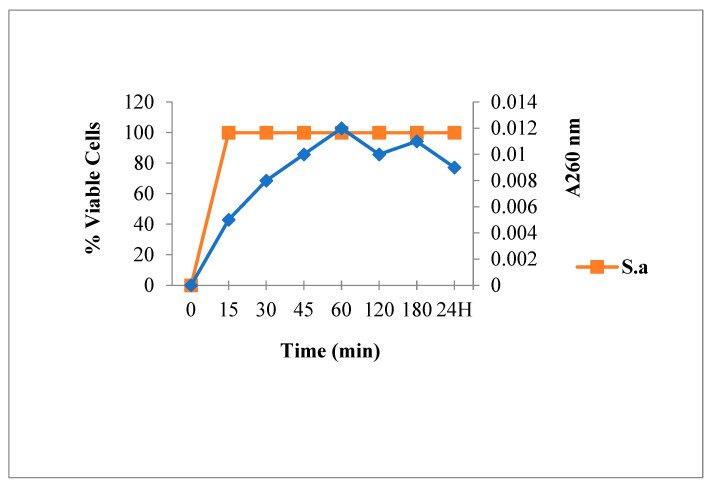
260 nm absorbent material release and viability of *S. aureus* in absence of antibacterial agent.

Bacteria viability and spontaneous lysis, without bactericidal agents, was verified under working conditions (Figure 2). Results showed neither mortality nor spontaneous cytoplasmic contents release. This suggested the integrity of the bacteria membrane.

**Figure 3:**
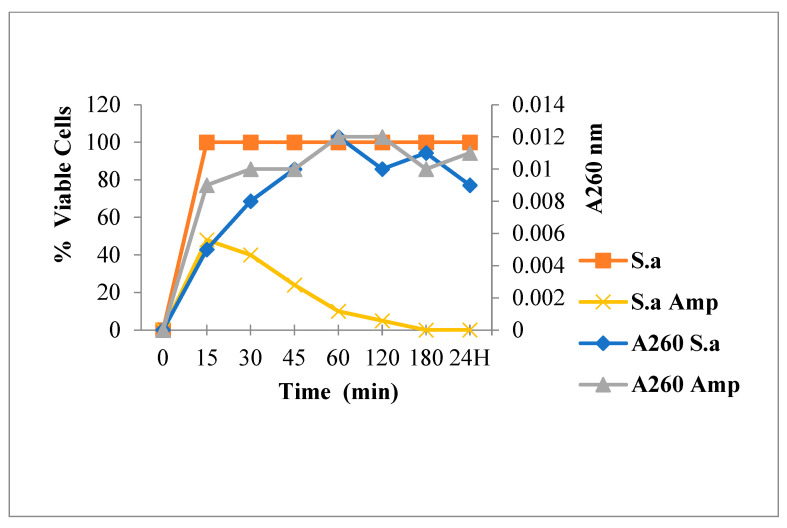
260 nm absorbent material release and viability of *S. aureus* following Ampicillin (**Amp**) treatment.

**Figure 4:**
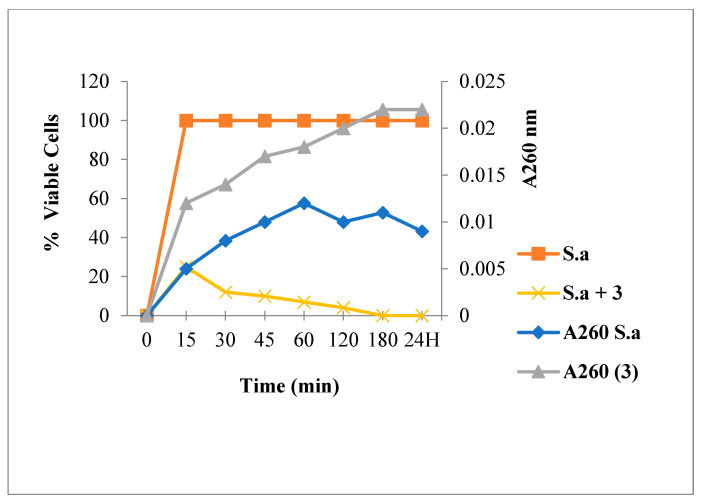
260 nm absorbent material release and viability of *S. aureus* following Chalcone **3** treatment.

**Figure 5:**
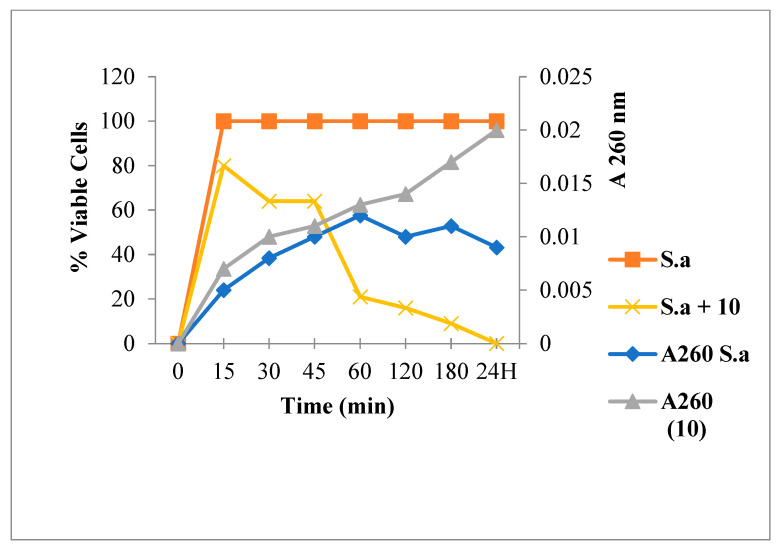
260 nm absorbent material release and viability of *S. aureus* following flavone **10** treatment.

**Figure 6:**
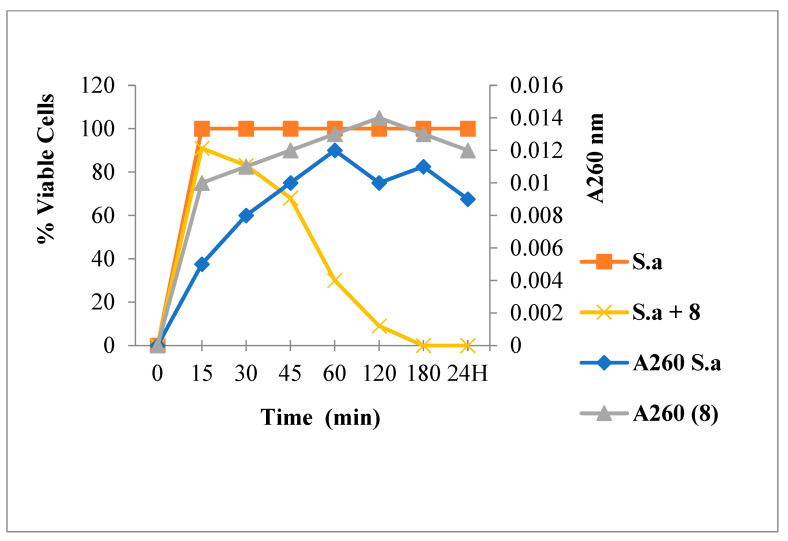
260 nm absorbent material release and viability of *S. aureus* following Flavanone **8** treatment.

Figure 3 shows that Amp treatment reduced absorbance at 260 nm and decreased the live bacterial cells number. As shown in Figure 4, Figure 5 and Figure 6, FLAV treatment induced an increase in absorbance at 260 nm with reduced bacterial viability. *S. aureus* mortality was more rapid following chalcone (**3**) exposure, suggesting a greater effect on the plasma membrane integrity.

#### 2.3.2. Nucleic Acid Release for *E. coli*

The nucleic acids amount of *E. coli* ATCC 8739 was estimated after treatment with a determined concentration (4 × [MIC]) of the most active FLAV; chalcone **4** (500 μg/mL)), flavone **11** (500 μg/mL), flavanone **8** (1000 μg/mL), and Colistin (8 μg/mL) by 260 nm absorbance measuring for 180 min and after 24 h.

**Figure 7:**
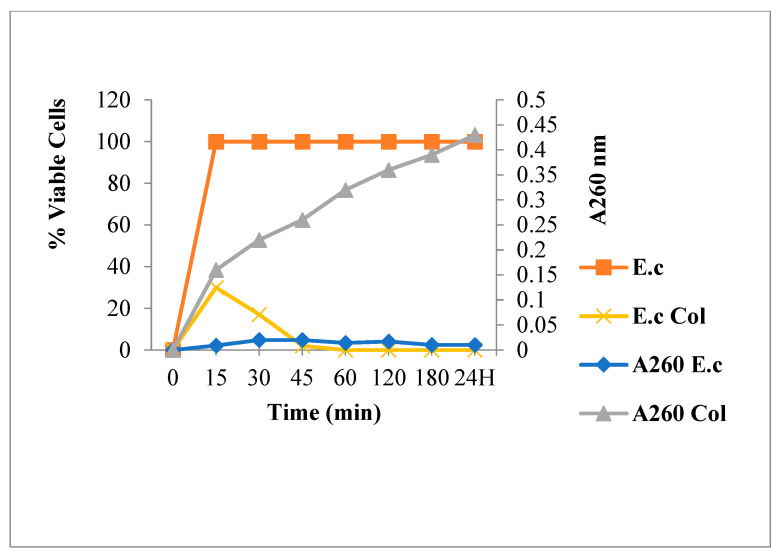
260 nm absorbent material release and *E. coli* viability following Colistin (**Col**) treatment.

Figure 7 highlights an increase in the bacterial content release at 260 nm with reduced living bacteria rate. The nucleic acids output started in the first few minutes of Colistin-bacteria contact and increased gradually over time, in parallel with a decrease in bacteria viability. The reduction begun in the first minutes of contact and ended with 100% of cell death. Belonging to the class of Polymyxins, Colistin alters and disrupts the cytoplasmic membrane permeability. This resulted in a periplasmic protein release in the first minutes, then the cellular constituents leakage after cytoplasmic membrane attack [42].

**Figure 8:**
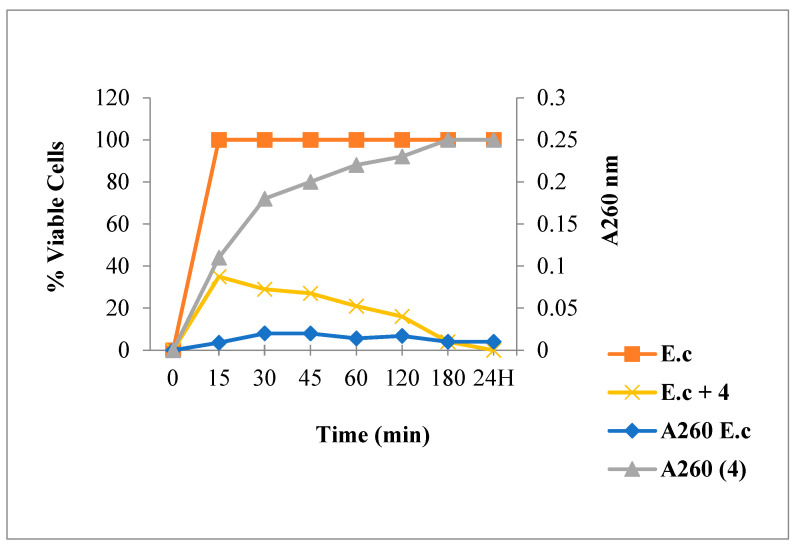
260 nm absorbent material release and *E. coli* viability following Chalcone **4** treatment.

**Figure 9:**
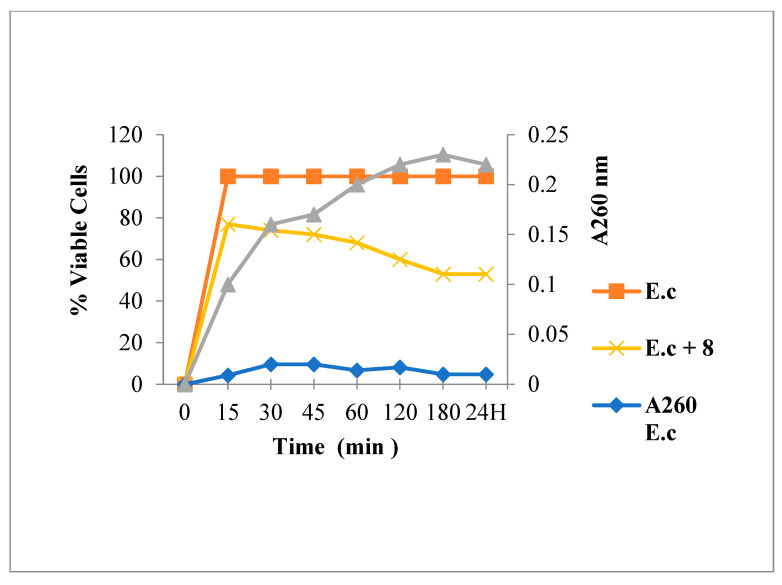
260 nm absorbent material release and *E. coli* viability following Flavanone **8** treatment.

**Figure 10:**
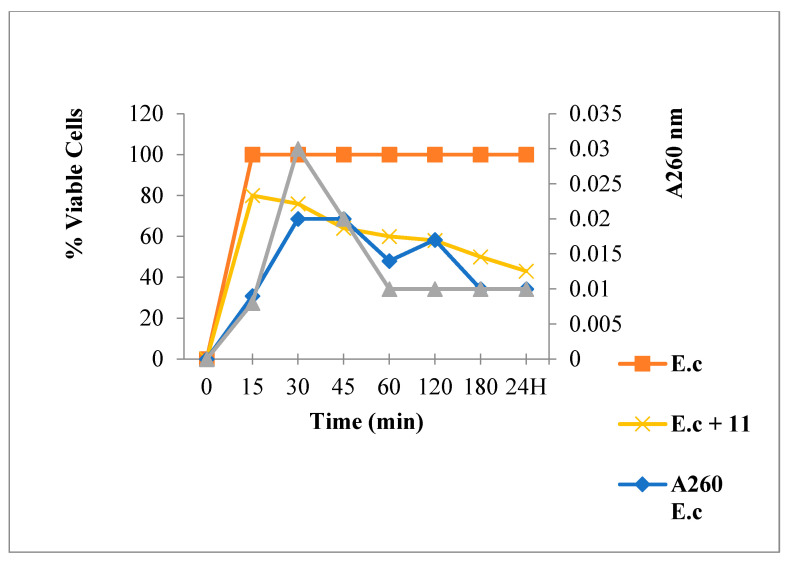
260 nm absorbent material release and *E. coli* viability following Flavone **11** treatment.

As is illustrated in Figure 8, Figure 9 and Figure 10, chalcone **4** and flavanone **8** treatments induced an absorbance increase and reduced the living bacteria rate while flavone **11** exposure showed low absorbance. Regarding the bactericidal effect, a total bacteria viability loss was observed from the first minutes of contact with the compound **4**.

**Figure 11:**
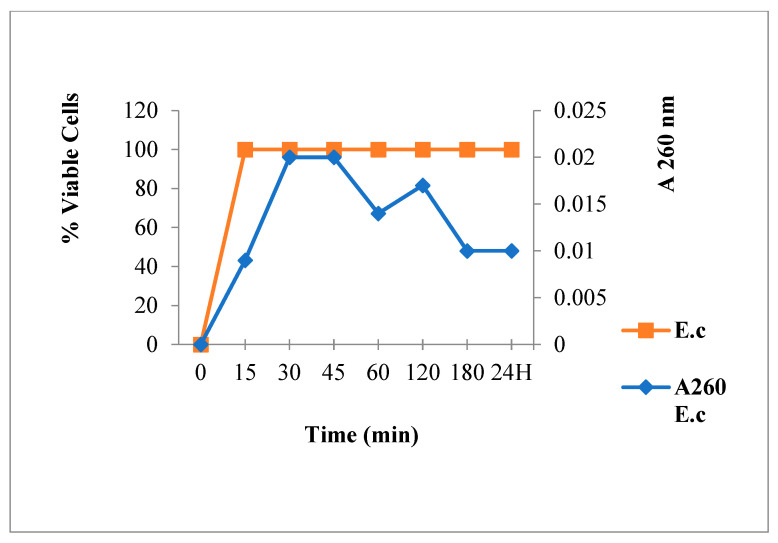
260 nm absorbent material release and viability of *E. coli* in absence of antibacterial agent.

The results also indicated a rapid initial release as well as significant mortality within the first few minutes of Colistin treatment and a progressive reduction of *E. coli* viability following various FLAV treatment compared to untreated cells (Figure 11). 

These findings also suggest that the substituted FLAV structures—the bromine group at the C6′ position for chalcones **3** and the methoxy group at the C4′ position for flavanones **8** are important for antibacterial activity against *S. aureus* 60 through a membrane permeabilization mechanism. This effect was suggested against *E. coli* ATCC 8739 for the C4′ methoxy substituted chalcone. These findings show that the FLAV antibacterial effect is linked to their structure, essentially to the B-ring substitutions. Our results are in agreement with previous studies; indeed, it has been reported that FLAV antimicrobial activity intensity is highly dependent on its chemical structure, which is particularly influenced by the number and position of various functional groups such as hydroxyl, methoxy, halogen, and methyl groups at the two aromatic rings (A and B) [43,44]. 

#### 2.3.3. Extraction and Purification of Nucleic Acids from Flavonoids Treated Bacteria

To check the effect of FLAV treatment on bacterial lysis and nucleic acid release, cells were treated with different FLAV at increasing concentrations (1, 2, 3, and 4 × MIC) and their nucleic acids were partially purified and analyzed by agarose gel electrophoresis.

**Figure 12:**
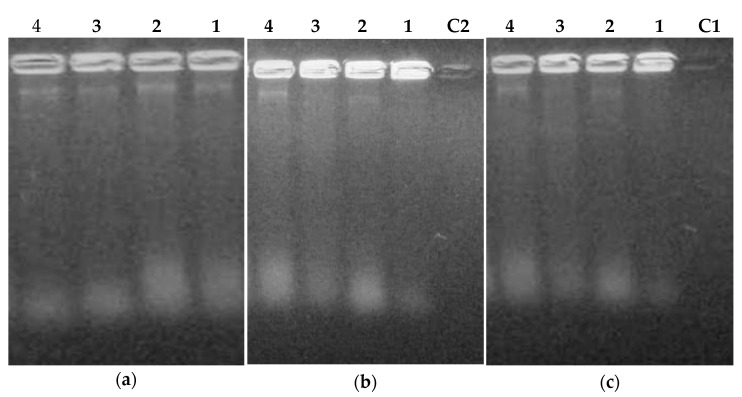
UV visualization of flavonoids treated *S.aureus* 60 lysates; (**a**) Chalcone **3** treatment, (**b**) Flavanone **8** treatment, (**c**) Flavone **10** treatment; **1** (1 × MIC) (µg/mL), **2** (2× MIC), **3** (3× MIC), **4** (4× MIC), **C2** (Amp), **C1** (Control).

The results presented in Figure 12 showed the presence of DNA bands and released RNA spots for the treated *S. aureus* cells with chalcone **3** and flavanone **8** molecules while flavone **10** exposure induced nucleic acids release only at a high concentration (4 × MIC). These results are in agreement with those obtained for the 260 nm absorbance evaluation and suggest a mechanism of bacterial lysis and membrane permeabilization under FLAV treatment.

**Figure 13:**
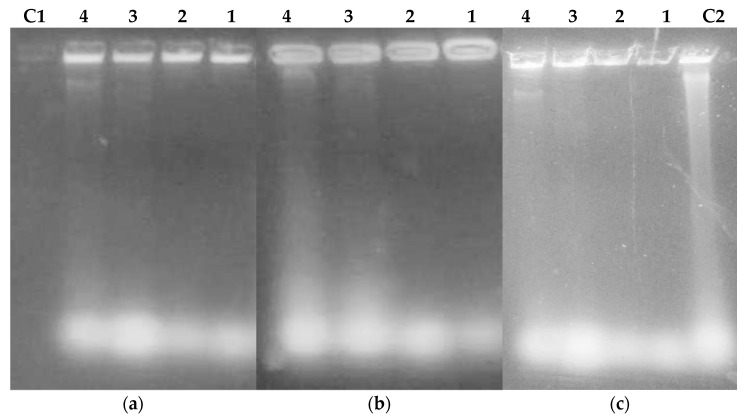
UV visualization of flavonoids treated *E. coli* ATCC 8739 lysates; (**a**) Chalcone **4** treatment, (**b**) Flavanone **8** treatment, (**c**) Flavone **11** treatment; **1** (1× MIC) (µg/mL), **2** (2× MIC), **3** (3× MIC), **4** (4× MIC), **C1** (Control), **C2** (Colistin).

For *E.coli* cells (Figure 13), DNA bands were only revealed at high concentrations for all the tested FLAV. This could be explained by the difference in cell structure between Gram-positive and Gram-negative bacteria which have an outer membrane consisting of lipopolysaccharides, phospholipids, and proteins conferring resistance [45].

#### 2.3.4. SEM Analysis, Cell Membrane Permeability and Morphological Changes

**Figure 14:**
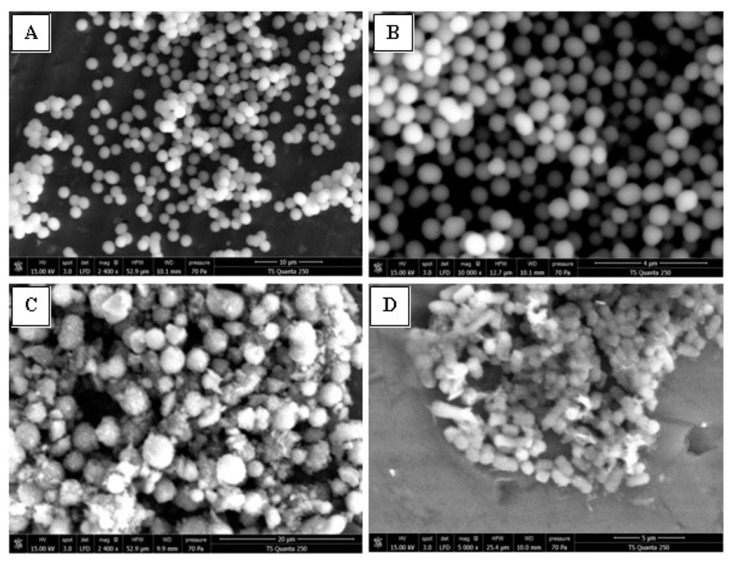
Scanning electron microscopy of untreated *S. aureus* (**A**) and *S. aureus* treated with 1 × MIC (**B**), 4 × MIC (**C**), and 10 × MIC (**D**) of flavonoids.

Figure 14 shows the SEM images of untreated (A) and treated (B–D) *S. aureus* 60 with the FLAV (**3**) at 1-fold (31.25 μg/mL), 4-fold (125 μg/mL), and 10-fold (312.5 μg/mL) MIC value, respectively. The images analysis clearly showed significant morphological damage of treated cells compared to the untreated control and sustains the hypothesis of membrane disruption. Untreated cells were rod shaped, regular, and intact (A) while FLAV treated cells became deformed, shriveled, and adhesive to each other (B–D). Moreover, the effect was dose dependent and more pronounced at 10-fold the MIC value. The chalcone also induced a bactericidal effect, resulting not only in the inhibition of bacterial growth but also in the killing of *S. aureus* 60 cells.

The effect of FLAV on cell membrane permeability of *S. aureus* was investigated on the basis of relative membrane integrity. As shown in our previous results, there was an obvious increase in the relative leaching out of 260 nm absorbent material and the presence of DNA bands and released RNA spots in the presence of various concentrations of FLAV with the increasing incubation time, indicating that the cell membrane integrity has been compromised after exposure of *S. aureus* to the tested chalcone. Furthermore, antibacterial action of FLAV against *S. aureus* was investigated using SEM to observe any morphological changes. As shown in Figure 14A, *S. aureus* cells in the absence of chalcone presented smooth, regular, and intact cell membrane structure. In comparison, a destructive effect of chalcone on *S. aureus* cells was observed after treatment with 4-fold and 10-fold MIC value (Figure 14C,D). Clearly, *S. aureus* cells became deformed and shriveled, which may lead to the leaching out of vital intracellular constituents and genetic materials. Moreover, the effect was dose dependent and more pronounced at 10-fold the MIC value. The chalcone also induced a bactericidal effect, resulting not only in the inhibition of bacterial growth but also in the killing of *S. aureus* cells.

Based on the current results it can be proposed that the antibacterial action of chalcone against *S. aureus* is targeting the cell membrane, causing a release of nucleic acids and even damaging the bacterial cell membrane. Similar results were reported for new tricyclic synthetic FLAV [23,46]. Moreover, Wang et al., 2017 [47], reported that thesechanges occurred in *S. aureus* when treated with MIC of 62.5 to 125 μg/mL of the *citrus* FLAV, Naringenin. 

This study enabled us to follow the cell content release in parallel with bacteria mortality and to show that FLAV activity targets the cell membrane and that chalcones could be promising molecules for the design of antibacterial agents against antimicrobial resistance developed by *S. aureus* species. 

The lipophilicity evaluation suggests that the FLAV bacterial membranes permeabilization capacity was mainly due to their lipophilic character. In fact, it is assumed that due to their lipophilic character, FLAV cross the cell membrane by passive diffusion, under their undissociated form, thus disrupting the cell membrane structure and possibly acidifying the cytoplasm and causing the bacterial cell contents leakage including proteins, nucleic acids, and inorganic ions such as potassium or phosphate [48]. In addition, according to Carson et al., 2002 [49], a marked cytoplasmic material leakage is considered to be indicative of global and irreversible cytoplasmic membrane damage. Additionally, this has been demonstrated by Mishra et al., 2009 [50]. Moreover, the FLAV antimicrobial activities depend mainly on the structure, their degree of hydroxylation, substitutions, and conjugations as well as their polymerization degree [51].

The correlation between antibacterial activity and membrane interference supports the theory that FLAV act by reducing the bacterial membrane fluidity. Haraguchi et al., 1998 [52] showed that licochalcone A inhibited radioactive precursors incorporation into macromolecules (DNA, RNA, and proteins) in *S. aureus* and *Micrococcus luteus*. This activity was similar to the respiratory chain mode of action. It has been suggested that the FLAV inhibition site was between CoQ and cytochrome of the bacterial cell electron transport chain.

## 3. Material and Methods

### 3.1. Microbial Strains Origin

Reference strains (*E. faecium* ATCC 19436, *S. aureus* ATCC 25923, *S. aureus* ATCC 6538, *B. cereus* ATCC 11778 and *E. coli* ATCC 8739) are from American Type Culture Collection, *P. aeruginosa,* MRSA 39, MSSA 60 are from the Laboratory of Microorganisms and Active Biomolecules Collection, University of Tunis El Manar, Tunis, Tunisia and *Salmonella* spp IPT13 is from Pasteur Institute of Tunis. Fungal strains *A. niger*, *A. flavus* and *P. expansum* are from the Laboratory of Microbial Ecology and Biotechnology, University of Paul Cezanne, France.

### 3.2. Antibacterial Activity

In vitro antibacterial activity of a series of chemically synthesized FLAV [53] was tested against various Gram-positive strains (*E. faecium* ATCC 19436, *S. aureus* ATCC 25923, *S. aureus* ATCC 6538, MRSA 39, MSSA 60 and *B. cereus* ATCC 11778) and Gram-negative (*E. coli* ATCC 8739, *P. aeruginosa,* and *Salmonella* spp IPT13) using Biolog’s Omnilog (BiologOmnilog^®^ Phenotype MicroArray™, Hayward, CA, USA) tool [54], which enables high throughput automated kinetic cell assays. The assays were conducted in 96-well microplates that can monitor chemical sensitivities. Cell response in each assay well is determined by the amount of color development produced by a tetrazolium compound reduction during cell respiration [55].

Colonies were harvested from the Brain Heart Infusion agar (BHIA) plate and suspended in BHI broth (BHIB). Bacterial suspension was then adjusted to 80–85% transmittance (T). The Mueller Hinton Broth (MHB, Biolife ItalianaS.r.I. viale Monza, 272-20128 Milano, Italy) with Biolog Redox Dye Mix (0.1%) was also prepared as control. FLAV stock solutions were two-fold serially diluted in Dimetylsulfoxyde (DMSO) in sterile 96 well microtiter plates containing the mixed (MHB-Biolog Redox Dye). The prepared cell suspension was then added in each well. 

Wells containing inoculum alone and inoculum with DMSO were used as negative controls. Standard ATB Amp and Van were used as positive controls. The plates were incubated at optimal temperature in the OmniLog plate incubator and reader and were monitored for color change. Readings were recorded automatically every 15 min for 18 to 24 h. Generated curves are compared to those of negative and positive controls and MICs were determined as the lowest concentrations for which there is no bacterial growth.

### 3.3. Antifungal Activity

*A. niger*, *A. flavus* and *P. expansum* were grown on Potato Dextrose Agar (PDA) plates at 28 °C. In order to prepare the control group, colonies were harvested from agar plate and suspended in NaCl solution (0.85%), and fungal suspension was adjusted to 62% transmittance (T). 

The Malt Extract (ME) (2%) with Biolog Redox Dye Mix was also prepared and FLAV stock solutions were two-fold serially diluted in DMSO in sterile 96 well microtiter plates, which contained the mixture ME-Biolog Redox Dye Mix. Later, fungal suspension was added. Wells containing inoculum alone and inoculum with DMSO were used as negative controls. Flu was used as a positive control. The microplates were incubated at 28 °C for 72 to 120 h in the OmniLog plate’s reader. Curves were compared to those of the controls and MICs were determined as the lowest concentrations for which there is no fungal growth.

### 3.4. Determination of Minimal Bactericidal Concentration (MBC) and Minimal Fungicidal Concentration (MFC)

The MBC and MFC of FLAV were determined according to the methods of Mbah et al. [56]. Briefly, 10 µL from wells corresponding to 1, 2, 3, and 4-fold of the MIC, were placed on a Nutrient Agar (GN) for bacteria and PDA (for fungi) and incubated at optimal temperatures for 24 h and 72h, respectively. MBC (/MFC) were defined as the lowest concentration with no bacterial or fungal growth.

### 3.5. Calcul of Lipophilicity

CLogP is a descriptor that is considered as an informative parameter of molecule solubility tendency. The higher the CLogP, the greater is the molecule lipophilicity but the lower is its tendencyto dissolve in the aqueous phase and conversely.Positive CLogP values indicate an increasing inhibitory activity [57]. CLogP was determined in silico by ChemDraw Professional 15.1 software; the chemical structure of each molecule is plotted on the software, which will generate the different physicochemical parameters including the LogP and CLogP values.

### 3.6. Mode of Action of Flavonoids

#### 3.6.1. Leakage of 260-nm Absorbing Material

Bacterial strains were cultured in Tryptic Soy Broth (TSB) and incubated at 37 °C for 18 h. After incubation, bacterial cells were harvested by centrifugation at 10,000× *g* for 10 min at 4 °C, then washed with Phosphate Buffered Saline (PBS); pH 7.4. Suspension concentrations were adjusted to approximately 10^9^ CFU ml^−1^. The most active FLAV (**10**, **3,** and **8** against *S. aureus* 60 and **11**, **4,** and **8** against *E. coli* ATCC 8739)) were added to the bacterial suspensions at four-fold of the MIC values. Suspensions were incubated at 35 °C and samples were removed at times 0, 15, 30, 45, 60, 120, and 180 min and after 24 h and centrifuged at 10,000× *g* for 10 min at 4 °C. 

Low molecular weights metabolites including nucleotides and their component structures (purines, pyrimidines, pentose, and inorganic phosphate), amino acids, and inorganic ions are known to leak from cells. Two hundred µL of supernatants from each condition were added to a 96-well plate (UV–transparent flat-bottom microplates, Costar Cat. No. 3635).

Wells and absorbance values at 260 nm were recorded using a UV spectrophotometer (Synergy HT, Bio-Tek, Highland Park, Winooski, VT, USA) by the method used by Nobmann et al., 2010 [58]. The following controls were included: a bacterial suspension in sterile PBS without antimicrobial agents as the negative control; a bacterial suspension with Amp for *S. aureus* 60 and with Colistin for *E. coli* ATCC 8739) as the positive controls.

#### 3.6.2. Bacterial Viability Determination

Bacterial suspensions collected after treatment with the different molecules (as described in Section 3.6.1) were serially diluted 10-fold in PBS. Aliquots of 100 µL were plated on nutrient agar and incubated at 37 °C for 24 h. The viability rate under FLAV and ATB treatments was calculated as the percentage of grown colonies compared to the untreated bacterial suspensions.

#### 3.6.3. Bacteria Lysis by Flavonoids, DNA, and RNA Extraction

Bacteria were cultured for 18 h at 37 °C in TSB, centrifuged for 1minat 4 °C (12,000× *g*), then they were washed in Tris-Glucose-EDTA (TGE) buffer and resuspended in the same buffer. Bacteria were then lysed by adding increasing concentrations of active FLAV. 

##### Extraction and Purification of Nucleic Acids

The obtained lysate was incubated for 30 min at 37 °C and 150 μL of 7.5 M ammonium acetate (pH 7.8) was added. Then, the lysate was placed on ice for 15 min to allow proteins, RNAs, and chromosomal DNA precipitation followed by harvesting for 10 min at 12,000× *g* at 4 °C. The nucleic acids were precipitated by adding ethanol (v/2v) for 30 min and then recovered by centrifugation for 15 min at 12,000× *g*, at 4 °C. Nucleic acid pellet was washed with 70% ethanol and dried for a few minutes. The pellet was dissolved in 50 µL of Tris-EDTA (TE) buffer and stored at −20 °C.

##### Agarose Gel Electrophoresis

Nucleic acids were mixed with a migration buffer-glycerol solution and then loaded on a 0.8% agarose gel in Tris-Acetate-EDTA (TAE) buffer. Electrophoresis was performed in TAE at 50 V until sufficient resolution was obtained. The bands were visualized by ethidium bromide in gel staining under UV light (254–366 nm).

#### 3.6.4. Scanning Electron Microscopy (SEM)

SEM was used to observe the morphological changes of FLAV-treated *S. aureus* 60. The bacterial cells obtained from the logarithmic growth phase were treated with the FLAV at 1, 4, and 10-fold of the MIC value at 37 °C for 18 h. Then, the suspensions were centrifuged at 12,000 rpm/min for 10 min. The sediments were washed with 0.1 M PBS, (pH=7.2) and fixed with 2.5% glutaraldehyde in PBS for 2 h at −4 °C. The cells were washed in the same buffer and were post-fixed for 30 min with osmium tetroxide. After harvesting, the cells were further dehydrated via graded ethanol concentrations (30%, 50%, 70%, 90%, and 100%) for 10 min each. Untreated bacterial cells were similarly processed and used as control. Then, cells were fixed on SEM support and observed by SEM (TS Quanta 250, Thermo Fischer Scientific, Hillsboro, OR, USA). The images were generated using a 15 kV electron beam.

## 4. Conclusions

A series of halogenated chalcones, flavanones, and flavones were evaluated for their in vitro antibacterial and antifungal activities. Most of the tested compounds inhibited the growth of both bacteria and fungi at lower concentrations. The antimicrobial activitywas more pronounced for chalcones towardGram-positive bacteria, in particular, *S. aureus* species. The effect of B-ring substitutions in the FLAV structure appears to be important for the antibacterial activity improvement, especially against *S. aureus* 60 and some SAR could be identified, such as free hydroxyl group in C2 for chalcone, halogens (bromine, chlorine) in C6′ and methoxy group in C4′. The good lipophilicity of studied FLAV also enhanced their antimicrobial effect on bacterial membrane, leading to its disruption and to the leakage of nucleic acids. This research showed that the studiedFLAV may constitute good candidates for the development of potent antimicrobial agents, especially the brominated chalcone, which could be a potential compound for the design of a membrane active drug against *S. aureus*. Therefore, further studies are still necessary to fully understand the involved mechanisms in order to elucidate the in vivo antibacterial activity.

## Figures and Tables

**Table 1:** MIC and MBC (μg/mL) of the tested flavonoids against Gram-negative bacteria.

	*E. coli*		*Salmonella* spp		*P. aeruginosa*	
Compound	MIC	MBC	MIC	MBC	MIC	MBC
**1**	250	250	125	500	125	500
**2**	250	250	125	≥500	125	≥500
**3**	250	250	250	≥500	125	500
**4**	125	125	250	500	125	≥500
**5**	≥500	≥500	500	>500	≥500	>500
**6**	250	≥500	125	500	250	≥500
**7**	500	≥500	500	>500	250	≥500
**8**	250	≥500	250	>500	125	≥500
**9**	250	500	125	≥500	250	≥500
**10**	250	500	250	≥500	250	≥500
**11**	125	500	250	≥500	125	500
**12**	250	500	≥500	≥500	250	≥500
**Amp**	≤3.9	≤3.9	62.5	62.5	500	>500
**Van**	250	250	250	250	250	250

*E. coli*: *Escherichia coli* ATCC 8739, *Salmonella* spp: *Salmonella* spp IPT13, *P. aeruginosa*: *Pseudomonas aeruginosa*, Amp: Ampicillin, Van: Vancomycin.

**Table 2:** MIC and MBC (μg/mL) of tested flavonoids against Gram-positive bacteria.

	*S. aureus* ^a^		*S. aureus* ^b^		*S. aureus* ^c^		*S. aureus* ^d^		*E. faecium*		*B. cereus*	
Cpd	MIC	MBC	MIC	MBC	MIC	MBC	MIC	MBC	MIC	MBC	MIC	MBC
**1**	125	250	62.5	250	125	>500	125	>500	125	>500	125	>500
**2**	62.5	250	62.5	62.5	125	500	31.25	125	62.5	500	31.25	>250
**3**	31.25	500	31.25	31.25	250	500	31.25	62.5	125	>500	125	500
**4**	62.5	≥500	62.5	125	250	>500	62.5	>500	125	>500	125	500
**5**	62.5	≥500	31.25	250	125	>500	125	1000	500	≥1000	125	500
**6**	62.5	500	31.25	250	500	>500	500	≥1000	500	≥1000	250	>500
**7**	62.5	250	15.62	≥125	500	>500	125	≥1000	500	≥1000	≥500	>500
**8**	62.5	250	15.62	≥125	125	>500	62.5	≥1000	62.5	≥500	62.5	>500
**9**	250	≥1000	62.5	≥500	250	>500	250	≥1000	250	≥1000	250	>500
**10**	31.25	≥250	31.25	250	≥ 500	>500	31.25	125	500	≥1000	500	500
**11**	125	1000	62.5	250	125	500	62.5	125	250	500	62.5	500
**12**	62.5	250	62.5	62.5	125	>500	62.5	≥500	250	250	62.5	500
**Amp**	≥500	>500	≤3.9	≤3.9	15.62	15.62	≤3.9	≤3.9	≥500	>500	250	250
**Van**	≤3.9	≤3.9	≤3.9	≤3.9	≤3.9	-	≤3.9	≤3.9	-	-	≤3.9	≤3.9

*S. aureus*^a^ 39: (MRSA), *S. aureus* ^b^ 60: (MSSA), *S. aureus* ^c^: *S. aureus* ATCC 6538, *S. aureus* ^d^: *S. aureus* ATCC 25923, *E. faecium*: *E. faecium* ATCC 19436, *B*. *cereus*: *B. cereus* ATCC 11778. Amp: Ampicillin, Van: Vancomycin.

**Table 3:** The antifungal activity of the tested flavonoids: MIC, MFC (μg/mL).

	*A. niger*		*A. flavus*		*P. expansum*	
Cpd	MIC	MFC	MIC	MFC	MIC	MFC
**1**	15.62	250	31.25	≥500	62.5	250
**2**	15.62	62.5	15.62	≥500	125	500
**3**	15.62	62.5	7.81	250	62.5	250
**4**	31.25	31.25	62.5	250	125	500
**5**	31.25	31.25	31.25	250	250	≥500
**6**	125	125	62.5	500	125	500
**7**	31.25	31.25	62.5	250	125	≥500
**8**	62.5	62.5	31.25	125	125	125
**9**	125	250	125	500	125	500
**10**	62.5	62.5	15.62	≥500	125	500
**11**	125	125	31.25	500	125	500
**12**	62.5	62.5	31.25	250	62.5	62.5
**Fluconazole**	62.5	62.5	62.5	62.5	7.81	7.81

**Table 4:** The lipophilicity (CLogP) of the tested flavonoids.

		LogP	CLogP
Chalcones	**1**	4.51	5.5296
	**2**	4.38	5.4486
	**3**	4.94	5.9099
	**4**	4.94	5.515
Flavanones	**5**	5.06	6.2426
	**6**	4.11	5.0469
	**7**	3.98	4.9659
	**8**	5.21	5.615
Flavones	**9**	5.33	6.3926
	**10**	4.67	5.7599
	**11**	4.38	5.052
	**12**	4.26	5.021

## Data Availability

Data concerning chemical synthesis of studied flavonoids are available at: https://journals.tubitak.gov.tr/chem/vol42/iss2/3 (accessed on 16 December 2022).

## References

[1] Ayukekbong J.A., Ntemgwa M., Atabe A.N. (2017). The threat of antimicrobial resistance in developing countries: Causes and control strategies. Antimicrob. Resist. Infect Contr..

[2] Paterson D.L. (2006). Resistance in Gram-negative bacteria: *Enterobacteriaceae*. Am. J. Infect. Control.

[3] Orhan D.D., Ozcelik B., Ozgen S., Ergun F. (2010). Antibacterial, antifungal, and antiviral activities of some flavonoids. Microbiol. Res..

[4] Magill S.S., Edwards J.R., Bamberg W., Zintars G.B., Dumyati G., Kainer M.A., Lynfield R., Maloney M., McAllister-Hollod L. (2014). Emerging Infections Program Healthcare-Associated Infections and Antimicrobial Use Prevalence Survey Team. Multistate point-prevalence survey of health care-associated infections. N. Engl. J. Med..

[5] Isenman H., Fisher D. (2016). Advances in prevention and treatment of vancomycin-resistant *Enterococcus* infection. Curr. Opin. Infect. Dis..

[6] Alanis A.L. (2005). Resistance to antibiotics: Are we in the post-antibiotic era?. Arch. Med. Res..

[7] Jubeh B., Breijyeh Z., Karaman R. (2020). Resistance of Gram-Positive Bacteria to Current Antibacterial Agents and Overcoming Approaches. Molecules.

[8] World Health Organization WHO Publishes List of Bacteria for Which Now Antibiotics are Urgently Needed. https://www.who.int/news/item/27-02-2017-who-publishes-list-of-bacteria-for-which-new-antibiotics-are-urgently-needed.

[9] Kakoullis L., Papachristodoulou E., Chra P., Panos G. (2021). Mechanisms of Antibiotic Resistance in Important Gram-Positive and Gram-Negative Pathogens and Novel Antibiotic Solutions. Antibiotics.

[10] Redgrave L.S., Sutton S.B., Webber M.A., Piddock L.J.V. (2014). Fluoroquinolone resistance: Mechanisms, impact on bacteria, and role in evolutionary success. Trends Microbiol..

[11] Liu Y.Y., Wang Y., Walsh T.R., Yi L.X., Zhang R., Spencer J., Doi Y., Tian G., Dong B., Huang X. (2016). Emergence of plasmid-mediated colistin resistance mechanism *mcr*-1 in animals and human beings in China: A microbiological and molecular biological study. Lancet Infect. Dis..

[12] Brink A.J. (2019). Epidemiology of carbapenem-resistant Gram-negative infections. Curr. Opin..

[13] Cushnie T.P.T., Lamb A.J. (2011). Recent advances in understanding the antibacterial properties of flavonoids. Int. J. Antimicrob. Agents.

[14] Okada K., Takamura Y., Yamamoto M., Inoue Y., Takagaki R., Takahashi K., Demizu S., Kajiyama K., Hiraga Y., Kinoshita T. (1989). Identification of antimicrobial and antioxidant constituents from *licorice* of Russian and Xinjiang origin. Chem. Pharm. Bull..

[15] Nowakowska Z. (2007). A review of anti-infective and anti-inflammatory chalcones. Eur. J. Med. Chem..

[16] Boubakeur B., Tirtouil A., Meddah B., Khadem H. (2015). The evaluation of the effect of synthetic flavonoids on growth of pathogenic and probiotic bacteria. J. Chem. Pharm. Res..

[17] Ohemeng K.A., Schwender C.F., Fu K.P., Barrett J.F. (1993). DNA gyrase inhibitory and antibacterial activity of some flavones. Bioorg. Med. Chem. Lett..

[18] Lal K., Yadav P., Kumar A., Kumar A., Paul A.K. (2018). Design, synthesis, characterization, antimicrobial evaluation and molecular modeling studies of some dehydroacetic acid-chalcone-1,2,3-triazole hybrids. Bioorg. Chem..

[19] Ayman M., El-Messery S.M., Habib E.E., Al-Rashood S.T., Almehizia A.A., Alkahtani H.M., Hassan G.S. (2019). Targeting microbial resistance: Synthesis, antibacterial evaluation, DNA binding and modeling study of new chalcone-based dithiocarbamate derivatives. Bioorg. Chem..

[20] Yadav P., Lal K., Kumar L., Kumar A., Kumar A., Paul A.K., Kumar R. (2018). Synthesis, crystal structure and antimicrobial potential of some fluorinated chalcone-1,2,3-triazole conjugates. Eur. J. Med. Chem..

[21] Tsuchiya H., Iinuma M. (2000). Reduction of membrane fluidity by antibacterial sophoraflavanone G isolated from *Sophora exigua*. Phytomedicine.

[22] Awolola G.V., Koorbanally N.A., Chenia H., Shode F.O., Baijnath H. (2014). Antibacterial and anti-biofilm activity of flavonoids and triterpenes isolated from the extracts of *Ficus Sansibarica warb*. Subsp. Sansibarica (Moraceae) extracts. Afr. J. Tradit. Complement. Altern. Med..

[23] Mori A., Nishino C., Enoki N., Tawata S. (1987). Antibacterial activity and mode of action of plant flavonoids against *Proteus vulgaris* and *Staphylococcus aureus*. Phytochemistry.

[24] Gupta V.K., Gaur R., Sharma A., Akther J., Saini M., Bhakuni R.S., Pathania R. (2019). A novel bifunctional chalcone inhibits multi-drug resistant Staphylococcus aureus and potentiates the activity of fluoroquinolones. Bioorg. Chem..

[25] Babii C., Mihalache G., Bahrin L.G., Neagu A.N., Gostin I., Mihai C.T., Sârbu L.G., Birsa L.M. (2018). A novel synthetic flavonoid with potent antibacterial properties: In vitro activity and proposed mode of action. PLoS ONE.

[26] Bano S., Javed K., Ahmad S., Rathish I.G., Singh S., Chaitanya M., Arunasree K.M., Alama M.S. (2013). Synthesis of some novel chalcones, flavanones and flavones and evaluation of their anti-inflammatory activity. Eur. J. Med. Chem..

[27] Farhadi F., Khameneh B., Iranshahi M., Iranshahy M. (2019). antibacterial activity of flavonoids and their structure–activity relationship: An update review. Phytother. Res..

[28] Elkanzi N.A.A., Hrichi H., Alolayan R.A., Derafa W., Zahou F.M., Bakr R.B. (2022). Synthesis of Chalcones Derivatives and Their Biological Activities: A Review. ACS Omega.

[29] Alcara´z L.E., Blanco S.E., Puig O.N. (2000). Antibacterial activity of flavonoids agains methicillin-resistant *Staphylococcus aureus* strains. J. Theor. Biol..

[30] Sahu N.K., Balbhadra S.S., Choudhary J., Kohli D.V. (2012). Exploring Pharmacological Significance of Chalcone Scaffold: A Review. Curr. Med. Chem..

[31] Kumar R., Mohanakrishnan D., Sharma A., Kaushik N.K., Kalia K., Sinha A.K., Sahal D. (2010). Reinvestigation of structure-activity relationship of methoxylated chalcones as antimalarials: Synthesis and evaluation of 2,4,5- trimethoxy substituted patterns as lead candidates derived from abundantly available natural -asarone. Eur. J. Med. Chem..

[32] Yang H.-M., Shin H.-R., Cho S.-H., Bang S.C., Song G.Y., Ju J.H., Kim M.K., Lee S.H., Ryu J.C., Kim Y. (2007). Structural requirement of chalcones for the inhibitory activity of interleukin-5. Bioorg. Med. Chem..

[33] Tran T.D., Do T.H., Tran N.C., Ngo T.D., Huynh T.N.P., Tran C.D., Thai K.M. (2012). Synthesis and anti Methicillin resistant *Staphylococcus aureus* activity of substituted chalcones alone and in combination with non-beta-lactam antibiotics. Bioorg. Med. Chem. Lett..

[34] Tran T.D., Nguyen T.T.N., Do T.H., Huynh T.N.P., Tran C.D., Thai K.M. (2012). Synthesis and Antibacterial Activity of Some Heterocyclic Chalcone Analogues Alone and in Combination with Antibiotics. Molecules.

[35] Husain A., Ahmad A., Mkhalid I.A.I., Mishra R., Rashid M. (2012). Synthesis and antimicrobial activity of bischalcone derivatives. Med. Chem. Res..

[36] Bahrin L.G., Hopf H., Jones P.G., Sarbu L.G., Babii C., Mihai A.C., Stefan M., Birsa L.M. (2016). Antibacterial structure–activity relationship studies of several tricyclic sulfur-containing flavonoids. Beilstein J. Org. Chem..

[37] Bahrin L.G., Sarbu L.G., Hopf H., Jones P.G., Babii C., Stefan M., Birsa M.L. (2016). The influence of halogen substituents on the biological properties of sulfur-containing flavonoids. Bioorg. Med. Chem..

[38] Bernini R., Pasqualetti M., Provenzano G., Tempesta S. (2015). Ecofriendly synthesis of halogenated flavonoids and evaluation of their antifungal activity. NJC.

[39] Mahalle P.R., Khaty N.T. (2010). Synthesis of Some Bromo-Substituted 3-Aroyl Flavanones and Flavones. E-J. Chem..

[40] Hasan A., Rasheed L., Malik A. (2007). Synthesis and characterization of variably halogenated chalcones and flavonols and their antifungal activity. As. J. Chem..

[41] Vasanthanathan P., Lakshmi M., Babu M.A., Gupta A.K., Kaskhedikar S.G. (2006). QSAR study of 3-Phenyl-5-acyloxymethyl -2H, 5H-furan-2ones as antifungal agents: The dominant role of electronic parameter. Chem. Pharm. Bull..

[42] Dixon R.A., Chopra I. (1986). Polymixin B and nonapeptide alter cytoplasmic membrane permeability in *Escherichia.coli*. J. Antimicrob. Chemother..

[43] Choi J.S., Chung H.Y., Kang S.S., Jung M.J., Kim J.W., Jung H.A. (2002). The structure–activity relationship of flavonoids as scavengers of peroxynitrite. Phytother. Res..

[44] Farkas O., Jakus J., Héberger K. (2004). Quantitative structure–antioxidant activity relationships of flavonoid compounds. Molecules.

[45] Liu Y., McKeever L.C., Malik N.S. (2017). Assessment of the antimicrobial activity of olive leaf extract against foodborne bacterial pathogens. Front. Microb..

[46] Babii C., Bahrin L.G., Neagu A.N., Gostin I., Mihasan M., Birsa L.M., Stefan M. (2016). Antibacterial activity and proposed action mechanism of a new class of synthetic tricyclic flavonoids. J. App. Microb..

[47] Wang L., Wang M., Zeng X., Xu X., Brennan C. (2017). Membrane and genomic DNA dual-targeting of *citrus* flavonoid 2 naringenin against *Staphylococcus aureus*. Integr. Biol..

[48] Campos F.M., Couto J.A., Figueiredo A.R., To I.V., Rangel A.O.S., Hogg T.A. (2009). Cell membrane damage induced by phenolic acids on wine lactic acid bacteria. Int. J. Food Microbiol..

[49] Carson C.F., Mee B.J., Riley T.V. (2002). Mechanism of action of *Melaleuca alternifolia* (tea tree) oil on *Staphylococcus aureus* determined by time-kill, lysis, leakage, and salt tolerance assays and electron microscopy. Antimicrob. Agents Chemother..

[50] Mishra A.K., Mishra A., Kehri K.H., Sharma B., Pandey A.K. (2009). Inhibitory activity of Indian spice plant *Cinnamomum zeylanicum* extracts against *Alternaria solani* and *Curvularia lunata*, the pathogenic dematiaceous moulds. Ann. Clin. Microbiol. Antimicrob..

[51] Kelly E.H., Anthony R.T., Dennis J.B. (2002). Flavonoid antioxidants: Chemistry, metabolism and structure-activity relationships. J. Nutr. Biochem..

[52] Haraguchi H., Tanimoto K., Tamura Y., Mizutani K., Kinoshita T. (1998). Mode of antibacterial action of retrochalcones from *Glycyrrhiza inflata*. Phytochemistry.

[53] Meddeb A., Bouhalleb G., Legros J., Rezgui F. (2018). Tetrahydronaphthalene as a precursor of new series of chalcones, flavanones and flavones. Turk. J. Chem..

[54] Biolog. https://www.biolog.com/products-portfolio-overview/phenotype-microarrays-for-microbial-cells/.

[55] Bochner B.R. (2003). New technologies to assess genotype-phenotype relationships. Nat. Rev. Genet..

[56] Mbah J., Ngemenya M.N., Abawah A., Babiaka S.B., Nubed L.N., Nyongbela K.D., Lemuh N., Efange S.M. (2012). Bioassay-guided discovery of antibacterial agents: In vitro screening of *Peperomia vulcanica*, *Peperomia fernandopoioana* and *Scleria striatinux*. Ann. Microbiol. Antimicrob..

[57] Wildman S.A., Crippen G.M. (1999). Prediction of physicochemical parameters by atomic contributions. J. Chem. Inf. Comput. Sci..

[58] Nobmann P., Bourke P., Dunne J., Henehan G. (2010). In vitro antimicrobial activity and mechanism of action of novel carbohydrate fatty acid derivatives against *staphylococcus aureus* MRSA. J. Appl. Microbiol..

